# The effect of 24 hours delay in oocyte maturation triggering in IVF/ICSI cycles with antagonist protocol and not-elevated progesterone: A randomized control trial 

**Published:** 2017-07

**Authors:** Robab Davar, Elham Naghshineh, Nosrat Neghab

**Affiliations:** 1 *Research and Clinical Center for Infertility, Yazd Reproductive Sciences Institute, Shahid Sadoughi University of Medical Sciences, Yazd, Iran.*; 2 *Department of Obstetrics and Gynecology, School of Medicine, Isfahan University of Medical Sciences, Isfahan, Iran.*

**Keywords:** Assisted reproductive technologies, In vitro fertilization, Randomized controlled trial

## Abstract

**Background:**

The best time of final oocyte maturation triggering in assisted reproduction technology protocols is unknown. This time always estimated by combined follicular size and blood progesterone level.

**Objective:**

The aim of this study was evaluation of the effect of delaying oocyte maturation triggering by 24 hr on the number of mature oocytes (MII) and other in vitro fertilization cycle characteristics in antagonist protocols with not-elevated progesterone (p ≤1 ng/ml).

**Materials and Methods:**

All patients' candidate for assisted reproduction technology underwent controlled ovarian hyperstimulation by antagonist protocol. When at least 3 follicles with ≥18 mm diameters were seen by vaginal ultrasonography; blood progesterone level was measured. The patients who had progesterone level ≤1 ng/dl entered the study. The participants' randomizations were done and patients were divided into two groups. In the first group, final oocyte maturation was done by human chorionic gonadotropin at the same day, but in the second group, this was performed 24 hr later. Oocytes retrieval was done 36 hr after human chorionic gonadotropin trigger by transvaginal ultrasound guide.

**Results:**

Number of retrieved oocytes, mature oocytes (MII), fertilized oocytes (2PN), embryos formation, number of transferred embryos and embryos quality has not significant differences between two groups. Also, fertilization and implantation rate, chemical and clinical pregnancy did not differ between groups.

**Conclusion:**

Delaying of triggering oocyte maturation by 24 hr in antagonist protocol with not-elevated progesterone (progesterone ≤1 ng/ml) have not beneficial nor harmful effect on the number of mature oocytes (MII) and other in vitro fertilization cycle characteristics.

## Introduction

There are limited information for appropriate time of final oocyte maturation triggering in assisted reproductive technology (ART) cycles (1). The best time for triggering final oocyte maturation is dependent on several factors, such as diameter of largest follicle, blood estradiol and progesterone level on trigger day, peak estradiol to the number of follicles larger than 14 mm ratio, and patient prior protocols used for controlled ovarian hyperstimulation. Oocyte quality and endometrium receptivity are depending on time of luteinization before oocyte retrieval (2). As mentioned by Hu *et al* that there is a positive relationship between follicular size and the level of cytoplasmic maturation. Postponing the oocyte maturation triggering through HCG administration delay, and so prolongation of follicular phase (which result in more mature oocytes) may have positive effects on clinical outcomes (2). 

Rise in blood progesterone level can be a predictor of follicles numbers and serum estradiol up rise (3). The negative impact of progesterone rise on the endometrium is more than the oocyte/embryo quality (1). High serum progesterone level could result in endometrial maturation progression and disable endometrial receptivity (4, 5). Van Vaerenbergh *et al*, Bosch *et al *and Labarta *et al* discovered that serum progesterone levels more than 1.5 ng/ml lead to transformation of gene expression in the endometrium, and cause reduced endometrial receptivity (6-8). Elevated serum progesterone levels on the day of HCG administration (p>1.5 ng/ml) decreased ongoing pregnancy and delivery rates (7, 9). Vandekerckhove *et al* showed, if the progesterone level is higher than 1 ng/ml, delaying the administration of HCG by 24 hr has no effect on the number of mature oocytes (10).

Although, Tremellen and Lane demonstrated that 24 hr delay in oocyte retrieval from the best predicted time can cause small but significant increase in collected oocytes numbers and embryos formation, but had no meaningful effect on live birth rates (11). Kolibianakis *et al* also represent that follicular phase lengthening by HCG delaying administration cause more advancement in endometrium on oocyte retrieval day (12).

Vandekerckhove *et al* showed that in patients with low serum progesterone level (≤1 ng/ml) 24 hr delaying in the oocyte maturation trigger could cause to more total oocytes and mainly more mature oocytes (MII) in contrast with those triggered on the same day. Although, no meaningful differences were seen in the pregnancy rates and pregnancy outcomes between two groups (10). But in the patients with higher serum progesterone level (>1 ng/ml) delaying the oocyte maturation trigger by 24 hr accompanied with no difference in IVF cycle characteristics (10). Venetis *et al*, Mio *et al* Bustillo *et al* and Ventis *et al* demonstrated in cycles with higher progesterone levels, more oocytes were retrieved, but a cutoff point for progesterone levels did not achieved (13-16). The best time for oocyte maturation triggering by use of the ultrasound criteria is still challengeable (17). Commonly, it is accepted that follicles must achieve to at least 17 mm in diameter before triggering for final oocyte maturation (18). 

The aim of this study was evaluation of the effect of 24 hr delaying of oocyte maturation triggering on the number of mature oocytes (MII) and other IVF cycle characteristics in antagonist cycles with not-elevated progesterone (p≤1 ng/ml).

## Materials and methods

This randomized controlled trial was performed at Research and Clinical Center for Infertility, Shahid Sadoughi University of Medical Sciences, Yazd, Iran, between August and October 2016. Based on Vandekerchhove *et al* study with Statistics Consultant, at least sample size of 20 patients in each group was needed (10). Infertile women, who their controlled ovarian hyperstimulation was done with GnRH antagonist protocol with fresh embryo transfer in the same cycle, were included. Participants with premature elevation of progesterone (>1 ng/ml) and non-fresh embryo transfer and sever male factor infertility were excluded. Eighty five women were included in the study (42 in the delay triggering group, and 43 in the same day triggering group). The patients were randomized by random allocation software. 

**Stimulation protocols**

All patients underwent GnRH antagonist controlled ovarian hyperstimulation protocols in ART cycles. The gonadotropins (Gonal-F, Merck-Serono, Spain) were started on day 2 of the menstrual cycle. The initial dose of gonadotropin was individualized for each infertile patient according to the age, body mass index, antral follicle count, anti-mulerian hormone (AMH) level, and previous responsiveness to ovarian stimulation. Then gonadotropins dose adjustments were done based on ovarian response by follicular diameter measurement with transvaginal ultrasound, obtained every 2 or 3 days from 7^th^ day of stimulation. 

The GnRH antagonist (cetrorelix, MERC-SERONO, France) was administered when the mean diameter of dominant follicles reached to 13-14 mm, and continues until the day of triggering with HCG. When at least 3 follicles with ≥18 mm diameter were seen by vaginal ultrasonography, then blood estradiol and progesterone level was measured. The patients who had progesterone level ≤1 ng/dl entered the study. The participants' randomizations were done using random numbers table, and patients were divided into two groups. 

In the first group, final oocyte maturation was done by HCG (Pregnyl, Organon, Netherlands) at the same day, and 24 hr later in the second group. Oocytes retrieval was performed 36 hr after HCG trigger through transvaginal ultrasound guided. Routine IVF/ICSI was performed as appropriate. The luteal phase was supported with vaginal progesterone (Cyclogest, Actavis, UK), 400 mg, twice a day. One or two embryos have transferred 2 days later. Fertilization rate was defined as the percentage of fertilized oocytes (2PN) to all mature oocytes (MII). Chemical pregnancies were confirmed 2 wk after embryo transfer, by positive serum HCG measurement. Clinical pregnancy was defined by the presence of gestational sac in the uterus, 4 wk after embryo transfer. The implantation rate was the proportion of embryos transferred resulting in an intrauterine gestational sac.

**Clinical data collection**

During treatment, all patients’ information including age, FSH level in 3^rd^ day of the menstrual cycle before the treatment, AMH, duration of gonadotropin administration, total gonadotropin dose, estradiol and progesterone levels on the HCG day, and number of total oocytes retrieved, number of mature oocytes (MII), and number of fertilized oocytes (2PN), numbers of embryo formation and transferred embryos were documented. Chemical and clinical pregnancy was identified, and fertilization rate and implantation rate were also calculated.

**Ethical consideration**

This study was approved by the Ethics Committee of the Shahid Sadoughi University of Medical Sciences (IR.SSU.RSI.REC. 1395.4). Informed written consent was provided from all participants.

**Statistical analysis**

The statistical analysis were performed using statistical package for the social sciences, version 17.0, (SPSS Inc, Chicago, Illinois, USA). Categorical data were expressed as number and percentage. For qualitative data, Chi- square test was used. For quantitative data, the normality status was checked at first with Kolmogrof Smirnoff test. In normal data distribution, student-t, and in abnormal data distribution, Mann whitney-U test was used.

## Results

**Figure 1:**
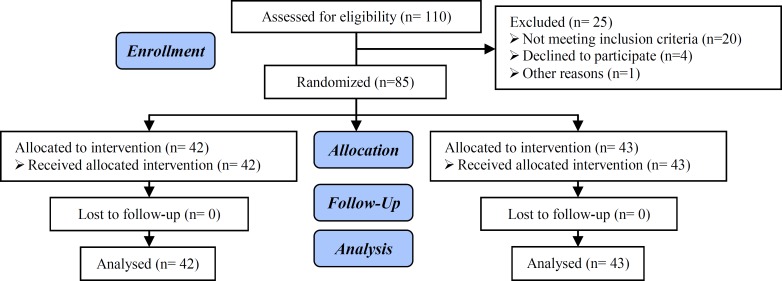
Consort flow chart

Totally 85 infertile women (42 in the delay triggering group, and 43 in the same day triggering group) participated in this study (Figure 1). The mean age of participants was 32.2±6.7 yr old (32.5±4.6 in the delay triggering group, and 31.9±4.7 in the same day triggering group). Other demographic and ART cycle characteristics are shown in table I and II. There were no significant differences in age, third day FSH, basal AMH, and other characteristics between two groups. Number of retrieved oocytes, mature oocytes (MII), fertilized oocytes (2PN), embryos formation, transferred embryos and embryos quality has not significant differences between two groups. Also, fertilization and implantation rate, chemical and clinical pregnancy did not differ between groups.

**Table I:** Demographic and basal characteristics of the participants

**Variable**		**Delay triggering group (n=42)**	**Same day triggering group (n=43)**	**p-value**
Age (years)^*^^#^		32.5 ± 4.6	31.9 ± 4.7	0.51
Infertility duration (years)^*^^$^		6.26 ± 0.59	6.98 ± 0.63	0.26
Infertility type^**^^&^				0.54
	Primary	33 (78.6%)	36 (83.7%)	
	Secondary	9 (21.4%)	7 (16.3%)	
No. of previous failed IVF^*^^$^		0.51 ± 0.19	0.50 ± 0.21	0.62
Third day FSH^*^^#^		6.1 ± 2.4	6.5 ± 2.7	0.66
AMH* ^#^		3.5 ± 3.7	3.4 ± 2.5	0.90

FSH= Follicle-stimulating hormone

AMH= Anti-Müllerian hormone

IVF: In vitro fertilization

* Data are presented as mean±SD

** Data are presented as n (%)

# Comparison was done by independent t-test

$ Comparison was done by Mann Whitney- U test

& Comparison was done by chi-square test

**Table II:** Cycle characteristics of the participants

**Variable**	**Delay triggering group (n=42)**	**Same day triggering group (n=43)**	**p-value**
Estradiol level on trigger day (pg/ml)^*^^#^	1547 ± 688	1841 ± 960	0.11
Progesterone level on trigger day (ng/ml)^*^^#^	0.72 ± 0.21	0.76 ± 0.16	0.28
Gonadotropin dose (IU)^*^^#^	2053 ± 666	2075 ± 821	0.89
Cycle duration (day)*^$^	14.2 ± 0.24	13 ± 0.30	0.03
Number of retrieved oocytes^*^^$^	9.5 ± 4.9	8.3 ± 4.7	0.22
Number of mature oocytes (MII) ^*^^$^	8.2 ± 0.66	7.5 ± 0.69	0.24
Number of 2PN ^*^^$^	4.6 ± 0.51	4.6 ± 0.56	0.73
Number of embryos ^*^^$^	4.1 ± 0.48	4.3 ± 0.57	0.82
Number of transferred embryos ^*^^$^	1.7 ± 0.13	1.9 ± 0.09	0.65
Total transferred embryo^$^	75	81	0.80
Embryo quality A^**^^&^	9 (12%)	8 (9.8%)	0.89
Embryo quality B ^**^^&^	41 (54.6%)	44 (54.3%)	
Embryo quality C ^**^^&^	25 (33.3%)	29 (35.8%)	
ET in trigger day (mm)^*^^e^	8.6 ± 0.22	9.0 ± 0.33	0.79
Fertilization rate	55%	59%	0.42
Chemical pregnancy ^**^^&^	11 (30.6%)	9 (23.7%)	0.34
Clinical pregnancy ^**^^&^	7 (19.4%)	8 (21.0%)	0.78
Implantation rate	10%	9.8%	0.91

pg/ml= Picogram/milliliter

ng/ml= Nanogram/milliliter

IU= International Unit

MII= Meiosis II (mature oocyte)

2PN= Two-pronuclear zygote

ET= Endometrial thickness

* Data are presented as mean±SD

** Data are presented as n (%)

$ Comparison was done by Mann Whitney- U test

& Comparison was done by chi-square test

## Discussion

The best time for oocyte maturation triggering by the ultrasound criteria is challengeable. In older cycles without use of GnRH agonist/antagonist, when maximum follicle diameter reached to 16 mm or more, and a serum estradiol was at least 600 pg/ml, triggering with hCG was considered (17). In antagonist cycles, the scientists agree with the leading follicle diameters of 16-17 mm (12, 19-21). In antagonist cycles, triggering is done earlier than in agonist cycles (21). In our study, we used follicle diameters (at least 3 follicles ≥ 18 mm) in selected patients with low progesterone levels (≤1 ng/ml). Numbers of retrieved oocytes, mature oocytes (MII), fertilized oocytes (2PN), embryos formation, transferred embryos and embryos quality have not significant differences between two groups (Table II). 

Vandekerckhove *et al* showed that in patients with low serum progesterone level (≤1 ng/ml), 24 hr delaying in the oocyte maturation trigger can cause more total oocytes and mainly more mature oocytes (MII), in contrast with those triggered on the same day (10). Tremellen and Lane demonstrated that 24 hr delay in oocyte retrieval can cause small but significant increase in collected oocytes numbers and embryos formation, but have no meaningful effect on live birth rates. They found that, advancing or delaying hCG administration by 1 day from ‘ideal’ had no adverse effect on IVF outcomes in GnRH antagonist cycles (11). 

Also, our study results demonstrated that 24 hr delay in triggering has no effect on fertilization and implantation rate, chemical and clinical pregnancy between two groups. Vandekerckhove *et al* advised that as soon as three follicles reached to a diameter ≥18 mm, decisions for best time of triggering must be made depend on the progesterone level. They recommended that if the progesterone level were more than 1 ng/ml, delaying in triggering has no effect on the mature oocytes. But if the progesterone level is ≤1 ng/ml, delaying oocyte maturation by 24 hr is advisable (9). Tremmelen and Lane found that advancing or delaying hCG administration by 1 day from ‘ideal’ had no adverse effect on IVF outcomes in GnRH antagonist cycles (10, 11). 

## Conclusion

Our findings in this study support these recommendations. Our study data showed that although delay in final oocyte triggering may have not beneficial effect on ART cycle's results, but it has not adverse effects, too.

## Note

***Registration ID in IRCT: IRCT2016071628756N3***
